# Supplementary material for: Degradation of CP4-EPSPS with a Psychrophilic Bacterium Stenotrophomonas maltophilia 780
Source: Biomolecules. 2022 Feb 17;12(2):318. doi: 10.3390/biom12020318 (PMC8869762; doi:10.3390/biom12020318)
Supplement: Supplementary file 1 [file biomolecules-12-00318-s001.zip › biomolecules-1574105-Supplementary Materials.pdf]

Supplementary information for “Degradation of CP4-EPSPS with a psychrophilic bacterium *Stenotrophomonas maltophilia* 780”

Figure S1. The sequence of CP4-EPSPS

5’TTTTGACGTTACCGACAGAATAAGCACCGGCTAACTCTGTGCCAGCAGCCGCGGTAATACAGAGGGTGCAAGCGT

TAATCGGAATTACTGGGCGTAAAGCGCGCGTAGGTGGTTTGTTAAGTTGGATGTGAAATCCCCGGGCTCAACCTGGG

AACTGCATCCAAAACTGGCAAGCTAGAGTATGGTAGAGGGTGGTGGAATTTCCTGTGTAGCGGTGAAATGCGTAGA

TATAGGAAGGAACACCAGTGGCGAAGGCGACCACCTGGACTGATACTGACACTGAGGTGCGAAAGCGTGGGGAGC

AAACAGGATTAGATACCCTGGTAGTCCACGCCGTAAACGATGTCAACTAGCCGTTGGGAGCCTTGAGCTCTTAGTGG

CGCAGCTAACGCATTAAGTTGACCGCCTGGGGAGTACGGCCGCAAGGTTAAAACTCAAATGAATTGACGGGGGCCC

GCACAAGCGGTGGAGCATGTGGTTTAATTCGAAGCAACGCGAAGAACCTTACCAGGCCTTGACATCCAATGAACTT

TCCAGAGATGGATTGGTGCCTTCGGGAACATTGAGACAGGTGCTGCATGGCTGTCGTCAGCTCGTGTCGTGAGATGT

TGGGTTAAGTCCCGTAACGAGCGCAACCCTTGTCCTTAGTTACCAGCACGTAATGGTGGGCACTCTAAGGAGACTGC

CGGTGACAAACCGGAGGAAGGTGGGGATGACGTCAAGTCATCATGGCCCTTACGGCCTGGGCTACACACGTGCTAC

AATGGTCGGTACAAAGGGTTGCCAAGCCGCGAGGTGGAGCTAATCCCATAAAACCGATCGTAGTCCGGATCGCAGT

CTGCAACTCGACTGCGTGAAGTCGGAATCGCTAGTAATCGCGAATCAGAATGTCGCGGTGAATACGTTCCCGGGCCT

TGTACACACCGCCCGTCACACCATGGGAGTGGGTTGCACCAGAAGTAGCTAGTCTAACCTTC-3’

Table S1. Strains obtained from CCTCC

Serial number	Molecular Taxonomy	Identity (%)	Geographical sources
CCTCC S2016780	*Mucilaginibacter gotjawali*	97.38	South Pole, Astronomical observatory
CCTCC S2016784	*Pseudomonas mandelii*	99.64	South Pole, Astronomical observatory
CCTCC S2016802	*Massilia eurypsychrophila*	99.36	South Pole, Astronomical observatory
CCTCC S2016808	*Arthrobacter oryzae*	99.49	South Pole, Astronomical observatory
CCTCC S2016809	*Pseudomonas chlororaphis*	98.39	South Pole, Astronomical observatory
CCTCC S2016709	*Massilia eurypsychrophila*	98.10	South Pole, the Larsemann Hills
CCTCC S2016713	*Arthrobacter oxydans*	98.45	South Pole, Inexpressible Island
CCTCC S2016714	*Arthrobacter globiformis*	98.15	South Pole, Inexpressible Island
CCTCC S2016723	*Arthrobacter oxydans*	98.73	South Pole, Inexpressible Island
CCTCC S2016730	*Arthrobacter oxydans*	98.66	South Pole, Inexpressible Island
CCTCC S2016741	*Pseudomonas marginalis*	99.7	South Pole, Larsemann Hills
CCTCC S2016742	*Pseudomonas marginalis*	99.7	South Pole, Larsemann Hills
CCTCC S2016746	*Sphingomonas oligophenolica*	98.6	South Pole, Larsemann Hills
CCTCC S2016754	*Brevundimonas intermedia*	99.69	South Pole, Larsemann Hills
CCTCC S2016766	*Sphingomonas asaccharolytica*	98.4	South Pole, Inexpressible Island
CCTCC S2016769	*Arthrobacter scleromae*	98.51	South Pole, Inexpressible Island
CCTCC S2016770	*Arthrobacter oxydans*	98.51	South Pole, Inexpressible Island
CCTCC S2016771	*Arthrobacter scleromae*	99.07	South Pole, Inexpressible Island
CCTCC S2016773	*Arthrobacter globiformis*	97.95	South Pole, Inexpressible Island
CCTCC S2016774	*Arthrobacter scleromae*	98.85	South Pole, Inexpressible Island
CCTCC S2016776	*Arthrobacter oxydans*	98.37	South Pole, Inexpressible Island

Figure S2. The 16S rDNA alignment of *Pseudomonas* sp. 780

Sequences producing significant alignments:

Select: All None Selected:0

Alignments Download GenBank Graphics Distance tree of results

Description	Max score	Total score	Query cover	E value	Perc. ident	Accession
Stenotrophomonas sp. strain D1 16S ribosomal RNA gene, partial sequence	2603	2603	100%	0.0	99.93%	MH814356.1
Stenotrophomonas sp. LY-2 gene for 16S ribosomal RNA, partial sequence	2603	2603	100%	0.0	99.93%	LC136883.1
Stenotrophomonas maltophilia strain 7K14 16S ribosomal RNA gene, partial sequence	2603	2603	100%	0.0	99.93%	KT825837.1
Stenotrophomonas maltophilia R551-3, complete genome	2603	10395	100%	0.0	99.93%	CP001111.1
Uncultured bacterium clone KRA30-21 16S ribosomal RNA gene, partial sequence	2603	2603	100%	0.0	99.93%	AY081992.1
Stenotrophomonas sp. 16S rRNA gene, isolate S3	2603	2603	100%	0.0	99.93%	AJ002814.1
Stenotrophomonas maltophilia strain JZFX-2 16S ribosomal RNA gene, partial sequence	2599	2599	99%	0.0	99.93%	MF170829.1
Bacterium strain YCR3A-3 16S ribosomal RNA gene, partial sequence	2599	2599	99%	0.0	99.93%	MF143454.1
Stenotrophomonas maltophilia strain SBR01 16S ribosomal RNA gene, partial sequence	2597	2597	100%	0.0	99.86%	KX018308.2
Stenotrophomonas maltophilia strain IARI-ABL-34 16S ribosomal RNA gene, partial sequence	2597	2597	100%	0.0	99.86%	KC581677.1
Stenotrophomonas sp. Bg1a partial 16S rRNA gene, isolate Bg1a	2597	2597	100%	0.0	99.86%	HF548402.1
Stenotrophomonas maltophilia strain KNUC2106 16S ribosomal RNA gene, partial sequence	2597	2597	100%	0.0	99.86%	JN084034.1
Uncultured bacterium clone 16slp101-2c06.p1k 16S ribosomal RNA gene, partial sequence	2597	2597	100%	0.0	99.86%	GQ157164.1
Uncultured bacterium clone 16slp101-3h04.p1k 16S ribosomal RNA gene, partial sequence	2597	2597	100%	0.0	99.86%	GQ157162.1
Uncultured bacterium clone 10A-13 16S ribosomal RNA gene, partial sequence	2597	2597	100%	0.0	99.86%	DQ906850.1

Tax BLAST report

Taxonomy report description

RID 9H7ZH7AE015 (Expires on 03-26 17:31 pm)

Query ID lcl|Query_97843
Description None
Molecule type nucleic acid
Query Length 1412

Database Name nr
Description Nucleotide collection (nt)
Program BLASTN 2.9.0+ ▷ Citation

Lineage Report

Organism Report Taxonomy Report

Organism	Blast Name	Score	Number of Hits	Description
Bacteria	bacteria		104	
. Stenotrophomonas	g-proteobacteria		68	
. . Stenotrophomonas sp.	g-proteobacteria	2603	9	Stenotrophomonas sp. hits
. . Stenotrophomonas sp. LY-2	g-proteobacteria	2603	1	Stenotrophomonas sp. LY-2 hits
. . Stenotrophomonas maltophilia	g-proteobacteria	2603	28	Stenotrophomonas maltophilia hits
. . Stenotrophomonas maltophilia R551-3	g-proteobacteria	2603	1	Stenotrophomonas maltophilia R551-3 hits
. . Stenotrophomonas sp. IARI-HHS2-27	g-proteobacteria	2603	1	Stenotrophomonas sp. IARI-HHS2-27 hits
Stenotrophomonas sp. KSB5	g-proteobacteria	2603	1	Stenotrophomonas sp. KSB5 hits

Figure S3.The 16S rDNA alignment of *S.maltophilia* 780

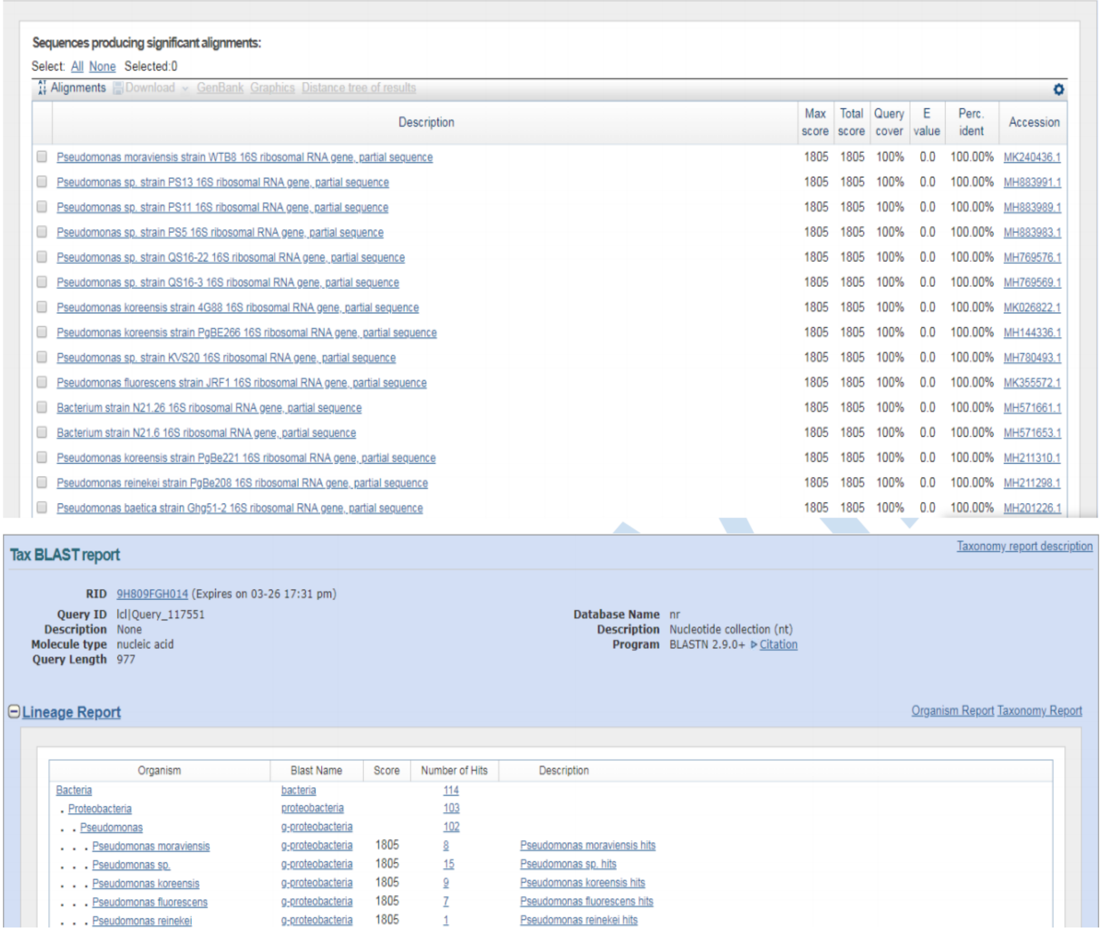
Sequences producing significant alignments:

Select: All None Selected:0

Alignments Download GenBank Graphics Distance tree of results

Description	Max score	Total score	Query cover	E value	Perc. ident	Accession
Pseudomonas moraviensis strain WTB8 16S ribosomal RNA gene, partial sequence	1805	1805	100%	0.0	100.00%	MK240436.1
Pseudomonas sp. strain PS13 16S ribosomal RNA gene, partial sequence	1805	1805	100%	0.0	100.00%	MH883991.1
Pseudomonas sp. strain PS11 16S ribosomal RNA gene, partial sequence	1805	1805	100%	0.0	100.00%	MH883989.1
Pseudomonas sp. strain PS5 16S ribosomal RNA gene, partial sequence	1805	1805	100%	0.0	100.00%	MH883983.1
Pseudomonas sp. strain QS16-22 16S ribosomal RNA gene, partial sequence	1805	1805	100%	0.0	100.00%	MH769576.1
Pseudomonas sp. strain QS16-3 16S ribosomal RNA gene, partial sequence	1805	1805	100%	0.0	100.00%	MH769569.1
Pseudomonas koreensis strain 4G88 16S ribosomal RNA gene, partial sequence	1805	1805	100%	0.0	100.00%	MK026822.1
Pseudomonas koreensis strain PgBE266 16S ribosomal RNA gene, partial sequence	1805	1805	100%	0.0	100.00%	MH144336.1
Pseudomonas sp. strain KVS20 16S ribosomal RNA gene, partial sequence	1805	1805	100%	0.0	100.00%	MH780493.1
Pseudomonas fluorescens strain JRF1 16S ribosomal RNA gene, partial sequence	1805	1805	100%	0.0	100.00%	MK355572.1
Bacterium strain N21.26 16S ribosomal RNA gene, partial sequence	1805	1805	100%	0.0	100.00%	MH571661.1
Bacterium strain N21.6 16S ribosomal RNA gene, partial sequence	1805	1805	100%	0.0	100.00%	MH571653.1
Pseudomonas koreensis strain PgBe221 16S ribosomal RNA gene, partial sequence	1805	1805	100%	0.0	100.00%	MH211310.1
Pseudomonas reinekei strain PgBe208 16S ribosomal RNA gene, partial sequence	1805	1805	100%	0.0	100.00%	MH211298.1
Pseudomonas baetica strain Ghg51-2 16S ribosomal RNA gene, partial sequence	1805	1805	100%	0.0	100.00%	MH201226.1

Tax BLAST report

Taxonomy report description

RID 9H809FGH014 (Expires on 03-26 17:31 pm)

Query ID lcl|Query_117551
Description None
Molecule type nucleic acid
Query Length 977

Database Name nr
Description Nucleotide collection (nt)
Program BLASTN 2.9.0+ Citation

Lineage Report

Organism Report Taxonomy Report

Organism	Blast Name	Score	Number of Hits	Description
Bacteria	bacteria		114	
. Proteobacteria	proteobacteria		103	
. . Pseudomonas	g-proteobacteria		102	
. . . Pseudomonas moraviensis	g-proteobacteria	1805	8	Pseudomonas moraviensis hits
. . . Pseudomonas sp.	g-proteobacteria	1805	15	Pseudomonas sp. hits
. . . Pseudomonas koreensis	g-proteobacteria	1805	9	Pseudomonas koreensis hits
. . . Pseudomonas fluorescens	g-proteobacteria	1805	7	Pseudomonas fluorescens hits
. . . Pseudomonas reinekei	g-proteobacteria	1805	1	Pseudomonas reinekei hits

Figure. S4 The time course of the degradation of the recombinant CP4-EPSPS with extracellular proteases of *Pseudomonas* sp. 780

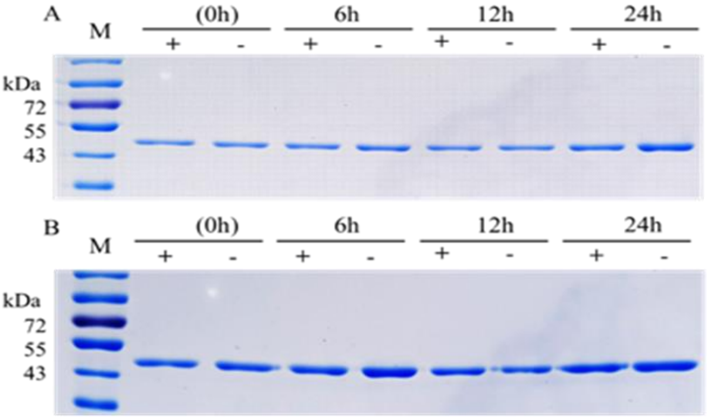

A. The degradation of 6×HIS-CP4-EPSPS; B. The degradation of CP4-EPSPS-6×HIS. (-): the negative control (degradation with supernatant of cell culture heated at 100°C for 30 min); (+) the extracellular proteases of *P.sp.* 780. M： protein molecular weight marker (the size of each band was indicated on the left).

Table S2. The putative ORFs coding proteases and peptidases in *S.maltophilia* 780

ID	E-value	Description
gi|692316381|ref|WP_032127221.1|	1.30E-124	peptidase [*Stenotrophomonas maltophilia*]
gi|501502945|ref|WP_012511135.1|	9.10E-301	peptidase S15 [*Stenotrophomonas maltophilia R551-3*]
gi|916992288|ref|WP_051599000.1|	0.00E+00	peptidase C39 [*Stenotrophomonas rhizophila*]
gi|504459148|ref|WP_014646250.1|	4.00E-290	peptidase M20 [*Stenotrophomonas maltophilia*]
gi|896534951|ref|WP_049437628.1|	9.70E-178	peptidase [*Stenotrophomonas maltophilia*]
gi|501501736|ref|WP_012509956.1|	0.00E+00	peptidase M14 carboxypeptidase A [*Stenotrophomonas maltophilia R551-3*]
gi|515925935|ref|WP_017356518.1|	0.00E+00	peptidase M13 [*Stenotrophomonas maltophilia*]
gi|501501696|ref|WP_012509917.1|	0.00E+00	peptidase M28 [*Stenotrophomonas maltophilia R551-3*]
gi|754363485|ref|WP_041864589.1|	1.10E-160	peptidase S41 [*Stenotrophomonas maltophilia*]
gi|896596053|ref|WP_049480189.1|	1.40E-232	Xaa-Pro aminopeptidase [*Stenotrophomonas maltophilia*]
gi|760006256|ref|WP_043689563.1|	8.10E-145	D-alanyl-D-alanine endopeptidase [*Luteimonas huabeiensis*]
gi|489189576|ref|WP_003098955.1|	1.30E-91	Lipoprotein signal peptidase [*Pseudomonas aeruginosa*]
gi|758873846|ref|WP_043034335.1|	4.50E-281	carboxypeptidase C (Cathepsin A) [*Stenotrophomonas maltophilia WJ66*]
gi|896525868|ref|WP_049429646.1|	0.00E+00	peptidase [*Stenotrophomonas maltophilia*]
gi|896504914|ref|WP_049412562.1|	0.00E+00	peptidase M20 [*Stenotrophomonas maltophilia*]
gi|491557240|ref|WP_005414827.1|	0.00E+00	Dipeptidyl peptidase IV [*Stenotrophomonas maltophilia EPM1*]
gi|896551079|ref|WP_049451894.1|	1.20E-233	metalloendopeptidase [*Stenotrophomonas maltophilia*]
gi|896545089|ref|WP_049446379.1|	3.40E-142	Methionine aminopeptidase [*Stenotrophomonas maltophilia*]
gi|896525822|ref|WP_049429600.1|	0.00E+00	peptidase [*Stenotrophomonas maltophilia*]
gi|475058159|emb|CCP15559.1|	7.90E-308	Plasma glutamate carboxypeptidase Flags: Precursor [*Stenotrophomonas maltophilia RA8*]
gi|493427208|ref|WP_006382890.1|	8.80E-151	Signal peptidase I [*Stenotrophomonas maltophilia RA8*]
gi|896551216|ref|WP_049451966.1|	1.60E-248	peptidase PmbA [*Stenotrophomonas maltophilia*]
gi|757859886|gb|KIS38398.1|	6.10E-102	endopeptidase YdhO [*Stenotrophomonas maltophilia WJ66*]
gi|922916199|gb|KOO79758.1|	0.00E+00	peptidase M13 [*Stenotrophomonas maltophilia*]

gi|612088648|gb|EZP47245.1|	0.00E+00	putative endopeptidase [*Stenotrophomonas sp. RIT309*]
gi|612085737|gb|EZP44404.1|	0.00E+00	Dipeptidyl-peptidase [*Stenotrophomonas sp. RIT309*]
gi|501503487|ref|WP_012511663.1|	1.40E-168	peptidase M48 Ste24p [*Stenotrophomonas maltophilia R551-3*]
gi|696407508|ref|WP_032978712.1|	2.80E-147	Peptidase [*Stenotrophomonas sp. RIT309*]
gi|815697551|ref|WP_046431082.1|	2.10E-172	peptidase [*Stenotrophomonas maltophilia*]
gi|501503155|ref|WP_012511339.1|	1.30E-92	peptidase A24A prepilin type IV [*Stenotrophomonas maltophilia R551-3*]
gi|498166821|ref|WP_010480977.1|	0.00E+00	peptidase S41 [*Stenotrophomonas maltophilia*]
gi|922919146|gb|KOO82692.1|	4.80E-181	peptidase [*Stenotrophomonas maltophilia*]
gi|759684756|ref|WP_043401703.1|	1.20E-98	peptidase [*Stenotrophomonas maltophilia*]
gi|501501432|ref|WP_012509656.1|	0.00E+00	peptidase M56 BlaR1 [*Stenotrophomonas maltophilia R551-3*]
gi|504458374|ref|WP_014645476.1|	1.10E-251	peptidase C69 [*Stenotrophomonas maltophilia*]
gi|740905942|ref|WP_038691190.1|	0.00E+00	dipeptidyl carboxypeptidase II [*Stenotrophomonas rhizophila*]
gi|896565148|ref|WP_049461671.1|	9.80E-113	signal peptidase I [*Stenotrophomonas maltophilia*]
gi|501503709|ref|WP_012511878.1|	1.90E-120	peptidase M22 glycoprotease [*Stenotrophomonas maltophilia R551-3*]
gi|501503686|ref|WP_012511856.1|	8.90E-160	Peptidase M23 [*Stenotrophomonas maltophilia R551-3*]
gi|194350216|gb|ACF53339.1|	6.30E-133	Serine-type D-Ala-D-Ala carboxypeptidase [*Stenotrophomonas maltophilia R551-3*]
gi|501501639|ref|WP_012509860.1|	3.60E-106	peptidase M15D vanX D-ala-D-ala dipeptidase [*Stenotrophomonas maltophilia R551-3*]
gi|490240882|ref|WP_004139140.1|	7.60E-270	Endopeptidase ykfC [*Stenotrophomonas maltophilia RA8*]
gi|896553556|ref|WP_049453837.1|	0.00E+00	peptidase S8 [*Stenotrophomonas maltophilia*]
gi|896549217|ref|WP_049450159.1|	6.10E-189	peptidase M23 [*Stenotrophomonas maltophilia*]
gi|922921281|gb|KOO84819.1|	2.10E-183	proline iminopeptidase [*Stenotrophomonas maltophilia*]
gi|501504211|ref|WP_012512377.1|	0.00E+00	peptidase M3A and M3B thimet/oligopeptidase F [*Stenotrophomonas maltophilia R551-3*]
gi|756166387|ref|WP_042614603.1|	8.90E-255	aminopeptidase [*Stenotrophomonas maltophilia*]
gi|647597629|ref|WP_025874228.1|	0.00E+00	peptidase [*Stenotrophomonas maltophilia*]

gi|692317689|ref|WP_032128510.1|	2.10E-147	peptidase [*Stenotrophomonas maltophilia*]
gi|692317678|ref|WP_032128499.1|	2.10E-231	peptidase M19 [*Stenotrophomonas maltophilia*]
gi|501501962|ref|WP_012510180.1|	6.90E-235	peptidyl-Asp metalloendopeptidase [*Stenotrophomonas maltophilia R551-3*]
gi|692318258|ref|WP_032129064.1|	0.00E+00	dipeptidyl-peptidase 7 [*Stenotrophomonas maltophilia*]
gi|815699437|ref|WP_046432751.1|	0.00E+00	endopeptidase IV [*Stenotrophomonas maltophilia*]
gi|896549036|ref|WP_049450010.1|	2.10E-270	cytosol aminopeptidase [*Stenotrophomonas maltophilia*]
gi|896524539|ref|WP_049428541.1|	0.00E+00	peptidase M61 [*Stenotrophomonas maltophilia*]
gi|896531560|ref|WP_049434638.1|	7.40E-98	peptidase A8 [*Stenotrophomonas maltophilia*]
gi|647600489|ref|WP_025877046.1|	6.60E-120	endopeptidase [*Stenotrophomonas maltophilia*]
gi|756165275|ref|WP_042613527.1|	0.00E+00	peptidase M20 [*Stenotrophomonas maltophilia*]
gi|475058091|emb|CCP15635.1|	1.30E-106	methionine aminopeptidase [*Stenotrophomonas maltophilia RA8*]
gi|493434413|ref|WP_006389956.1|	4.80E-106	LON peptidase N-terminal domain and RING finger protein 2 [*Stenotrophomonas maltophilia RA8*]
gi|896545772|ref|WP_049447062.1|	2.50E-145	peptidase [*Stenotrophomonas maltophilia*]
gi|896565889|ref|WP_049462250.1|	0.00E+00	peptidase S46 [*Stenotrophomonas maltophilia*]
gi|896509486|ref|WP_049416816.1|	4.70E-258	proline dipeptidase [*Stenotrophomonas maltophilia*]
gi|503804357|ref|WP_014038351.1|	1.30E-252	peptidase M24B X-Pro dipeptidase/aminopeptidase domain protein [*Stenotrophomonas maltophilia JV3*]
gi|653607042|ref|WP_027618358.1|	4.80E-127	peptidase S24 [*Pseudomonas sp. URHB0015*]
gi|491553502|ref|WP_005411093.1|	9.50E-96	peptidase [*Stenotrophomonas maltophilia*]
gi|756166096|ref|WP_042614321.1|	0.00E+00	peptidase [*Stenotrophomonas maltophilia*]
gi|922683161|gb|ALA83957.1|	0.00E+00	prolyl oligopeptidase [*Stenotrophomonas maltophilia*]
gi|515923736|ref|WP_017354319.1|	9.50E-213	D-alanyl-D-alanine carboxypeptidase [*Stenotrophomonas maltophilia*]
gi|756166822|ref|WP_042615018.1|	0.00E+00	aminopeptidase [*Stenotrophomonas maltophilia*]
gi|922682389|gb|ALA83185.1|	0.00E+00	dipeptidyl carboxypeptidase II [*Stenotrophomonas maltophilia*]
gi|692318908|ref|WP_032129696.1|	0.00E+00	aminoacyl peptidase [*Stenotrophomonas maltophilia*]
gi|501504117|ref|WP_012512283.1|	2.70E-281	peptidase M56 BlaR1 [*Stenotrophomonas maltophilia R551-3*]

gi|896542396|ref|WP_049443938.1|	0.00E+00	oligopeptidase A [*Stenotrophomonas maltophilia*]
gi|896493993|ref|WP_049402634.1|	6.10E-262	peptidase S41 [*Stenotrophomonas maltophilia*]
gi|503801718|ref|WP_014035712.1|	0.00E+00	peptidase M28 [*Stenotrophomonas maltophilia JV3*]
gi|501501832|ref|WP_012510051.1|	5.90E-178	Peptidase M23 [*Stenotrophomonas maltophilia R551-3*]
gi|917542362|ref|WP_052148779.1|	1.50E-181	proline iminopeptidase [*Stenotrophomonas maltophilia*]
gi|566142589|ref|WP_024011406.1|	1.60E-10	peptidase [*Pseudomonas moraviensis R28-S*]
gi|917755073|ref|WP_052269126.1|	1.70E-200	peptidase M42 [*Pseudomonas fluorescens*]
gi|493413088|ref|WP_006369023.1|	9.60E-215	Uncharacterized metalloprotease [*Stenotrophomonas maltophilia RA8*]
gi|493412491|ref|WP_006368434.1|	0.00E+00	protease domain-containing protein [*Stenotrophomonas maltophilia RA8*]
gi|746376602|ref|WP_039420382.1|	1.00E-169	serine protease [*Xanthomonas vesicatoria*]
gi|922918882|gb|KOO82428.1|	0.00E+00	Clp protease ClpC [*Stenotrophomonas maltophilia*]
gi|896276656|ref|WP_049263841.1|	1.10E-179	Clp protease ClpC, partial [*Pseudomonas aeruginosa*]
gi|758873988|ref|WP_043034476.1|	1.30E-275	cysteine protease [*Stenotrophomonas maltophilia*]
gi|896505601|ref|WP_049413215.1|	1.70E-200	stomatin/prohibitin-family membrane protease subunit [*Stenotrophomonas maltophilia*]
gi|922682618|gb|ALA83414.1|	1.20E-267	protease TldD [*Stenotrophomonas maltophilia*]
gi|872686407|emb|CRQ79460.1|	5.20E-307	protease TldD [*Pseudomonas aeruginosa*]
gi|696406882|ref|WP_032978097.1|	0.00E+00	Zinc protease [*Stenotrophomonas sp. RIT309*]
gi|493410619|ref|WP_006366596.1|	8.80E-57	ATP-dependent Clp protease adapter protein clpS [*Stenotrophomonas maltophilia RA8*]
gi|740903143|ref|WP_038688391.1|	1.30E-147	Clp protease ClpX [*Stenotrophomonas rhizophila*]
gi|501503709|ref|WP_012511878.1|	1.90E-120	peptidase M22 glycoprotease [*Stenotrophomonas maltophilia R551-3*]
gi|515926406|ref|WP_017356989.1|	2.50E-41	ATP-dependent Clp protease proteolytic subunit [*Stenotrophomonas maltophilia*]
gi|495543884|ref|WP_008268463.1|	6.20E-300	protease [*Stenotrophomonas sp. SKA14*]
gi|922686726|gb|ALA91477.1|	6.40E-155	membane protease HflC [*Stenotrophomonas maltophilia*]

gi|504459393|ref|WP_014646495.1|	6.80E-252	zinc metalloprotease [*Stenotrophomonas maltophilia*]
gi|872681110|emb|CRR57107.1|	1.90E-111	Protease synthase and sporulation protein PAI 2 [*Pseudomonas aeruginosa*]
gi|872680093|emb|CRR70935.1|	1.80E-117	Protease production enhancer protein [*Pseudomonas aeruginosa*]
gi|740576582|ref|WP_038362377.1|	3.10E-205	Clp protease ClpP [*Pseudomonas chlororaphis*]
gi|493437803|ref|WP_006393294.1|	1.10E-216	ATP-dependent protease ATP-binding subunit HslU [*Stenotrophomonas maltophilia RA8*]
gi|518167463|ref|WP_019337671.1|	9.70E-16	ATP-dependent protease [*Stenotrophomonas maltophilia*]
gi|501503639|ref|WP_012511810.1|	4.10E-290	protease Do [*Stenotrophomonas maltophilia R551-3*]
gi|490254907|ref|WP_004152871.1|	7.00E-134	cell division protease FtsH [*Stenotrophomonas maltophilia RA8*]
gi|748748842|ref|WP_040006958.1|	0.00E+00	serine protease [*Stenotrophomonas sp. SKA14*]
gi|219719191|gb|EED37716.1|	4.80E-106	ATP-dependent zinc-metallo protease [*Stenotrophomonas* sp. *SKA14*]
gi|647601653|ref|WP_025878144.1|	3.00E-121	Lon protease [*Stenotrophomonas sp. RIT309*]
gi|490248191|ref|WP_004146319.1|	1.00E-100	ATP-dependent protease ATP-binding subunit ClpX [*Stenotrophomonas maltophilia RA8*]
gi|748192953|ref|WP_039764045.1|	8.60E-20	Clp protease [*Pseudomonas fluorescens*]
gi|734951378|ref|WP_034151555.1|	3.50E-48	serine protease [*Pseudomonas fluorescens*]
